# Massive online data annotation, crowdsourcing to generate high quality sleep spindle annotations from EEG data

**DOI:** 10.1038/s41597-020-0533-4

**Published:** 2020-06-19

**Authors:** Karine Lacourse, Ben Yetton, Sara Mednick, Simon C. Warby

**Affiliations:** 1 Centre d’études avancées en médecine du sommeil, Montréal, Canada; 2 Department of Cognitive Science, University of California, Irvine, CA USA; 3 Department of Psychiatry, Université de Montréal, Montréal, Canada

**Keywords:** Computational neuroscience, Non-REM sleep, Predictive markers

## Abstract

Spindle event detection is a key component in analyzing human sleep. However, detection of these oscillatory patterns by experts is time consuming and costly. Automated detection algorithms are cost efficient and reproducible but require robust datasets to be trained and validated. Using the MODA (Massive Online Data Annotation) platform, we used crowdsourcing to produce a large open-source dataset of high quality, human-scored sleep spindles (5342 spindles, from 180 subjects). We evaluated the performance of three subtype scorers: “experts, researchers and non-experts”, as well as 7 previously published spindle detection algorithms. Our findings show that only two algorithms had performance scores similar to human experts. Furthermore, the human scorers agreed on the average spindle characteristics (density, duration and amplitude), but there were significant age and sex differences (also observed in the set of detected spindles). This study demonstrates how the MODA platform can be used to generate a highly valid open source standardized dataset for researchers to train, validate and compare automated detectors of biological signals such as the EEG.

## Introduction

Sleep spindles are brief 10–16 Hz bursts of brain activity during stage N2 and N3 sleep. They are typically recorded from cortical surfaces by electroencephalography (EEG) and are markers of sleep dependent cognition^1^, early indicators of mental disorders^2^ or brain deterioration due to age^3^. Spindles follow a characteristic waxing and waning profile, and generally last 0.5 to 1.0 seconds in duration. These characteristics are predominately trait-like, and remain remarkably stable night after night within an individual, but vary between individuals^4^. A small but consistently observed decrease of the spindle density, amplitude and duration occurs with age^5–9^. Sex differences of spindle activity linked to memory or aging have been reported^10–13^, where women tend to be less affected by aging^6,10^ resulting a greater spindle activity (peak-to-peak amplitude^14^ and density^4,7^) in women than men, particularly in the elderly. Characteristics of spindles may index the underlying neuroanatomy involved in normal brain function, particularly in the processing of learning and memory, and have been related to intelligence^15–21^.

As well as their relation to biological processes, the detection of spindles is a key component in analyzing human sleep, as spindles are used to indicate the transition from stage N1 to N2 sleep during sleep scoring. However, detection and quantification of these oscillatory patterns by highly trained experts is time consuming and costly. Further, the definition of sleep spindles(A train of distinct waves with frequency 11–16 Hz with a duration > = 0.5 seconds, usually maximal in amplitude using central derivations)^22^ is not entirely precise, and experts disagree on variations of sleep spindles. As well, the EEG signal may be obscured by other signal phenomena, thereby limited human detection. Critical for the advancement of sleep science is the development of automated feature detection tools. Recent years have highlighted the power of machine learning methods in the biosciences to augment expert clinical judgment. For example, cardiologist level arrhythmia detection^23^, or seizure diagnosis^24^. Automated methods do not fatigue, are cost efficient, remain consistent, and are readily deployable. However, previous studies have suggested that there are important differences between human and algorithm detected spindles^14^, leading to conflicting results depending on how spindles were detected^25,26^. For instance, a significant decrease in sleep spindle density using visual scoring was observed in autism patients^27–29^, whereas an increase or no difference was found using an automated detector^30,31^. Similarly, in narcolepsy, a decrease of spindle density was observed with visual scoring^32^ but not replicated with an automated algorithm^33^. While automated methods show great promise for sleep science, they require large, highly valid datasets, which were not previously available. Here we introduce a large, open, highly valid dataset of human sleep spindles collected through crowdsourcing.

Crowdsourcing, which has been previously used to collect spindle data^14,34,35^, involves collating the judgments of a large number of human scorers to reach a high quality “gold standard” consensus. This data collection method leverages the “wisdom of the crowd” effect^36^, where the collective opinion of a group of individuals tends to be more accurate than a single expert. Crowdsourcing yields better spindle detection, and captures more generalizable spindle properties than single expert scoring because each scorer contributes only little, thereby reducing errors from fatigue and distraction, and we capture a diverse, unbiased opinion on what represents a true spindle, which is especially important given the imperfect agreement between experts. The idea of crowdsourcing for sleep science was first introduced by Warby *et al*.^14^, where segments of stage 2 sleep (from 110 subjects) were viewed by a mean of 5 experts and 11 non-expert Mechanical Turk (mturk) Workers. Agreement between experts (average individual f1 = 0.67 against gold standard) and the performance of the group consensus of non-experts against the gold standard (f1 = 0.67) were high, and non-experts outperformed the automated detectors. Unfortunately, due to privacy concerns, the polysomnography dataset used in this study is not openly available to the public, greatly restricting its use as a benchmark for algorithm validation. Ray *et al*.^9^ independently developed a similar paradigm to Warby *et al*.^14^ but used the openly available Montreal Archive of Sleep Studies (MASS)^37^. Each segment of EEG (from 15 subjects) was viewed by two experts and a mean of 18 non-expert mturk workers. Agreement between the non-expert consensus and the expert who scored in similar conditions than the non-experts was substantial (f1 = 0.81), but a moderate agreement was observed between the only two experts who scored MASS (f1 = 0.54), limiting the validity of the expert dataset of spindles. Similarly Zhao *et al*.^35^ collected spindles scoring in a crowdsourcing scheme from 5 experts and 168 non-experts (at least 20 non-experts per segment) and reported a high agreement between the non-expert and expert consensus (f1 = 0.78), unfortunately the dataset used is not open source. We aimed to build upon the success of these three studies and produce a large, open dataset of high quality spindles from both young and old subjects. Using this dataset, we ask: a) Can many non-experts match the quality of an expert technician with much lower cost and completion time? b) Do experts agree on spindle features, and if so, what are they? c) How do spindle features change across age and sex? Further, the conclusions that drive sleep science are often built upon spindles scored by non-technician researchers. Therefore, we added a non-PSG-tech “researcher” group, composed of graduate students, postdocs and faculty in the sleep science field and compare these to formally trained PSG experts.

To facilitate scoring, we developed a web-based open source online scoring platform, named MODA for Massive Online Data Annotation. The MODA platform allowed scorers from around the world to perform the spindle-identification tasks wherever and whenever they chose. While, in this study, we have used MODA for spindle scoring, it is an adaptable platform that could be easily used for the crowdsourced scoring of any EEG or biosignal-based annotation task. In this paper, we described how data was crowdsourced and analyzed. A number of Group Consensuses (GCs) were created by aggregating the scoring of many scorers, thereby removing idiosyncratic noise and increasing validity of the spindle dataset. GCs in this study were compiled from the three different user subtypes independently: PSG technologists (experts; *exp*), researchers (*re*) and non-experts (*ne*). The PSG technologists, who are trained and perform spindle scoring regularly as part of their work, are considered the experts, and their GC is designated the formal and highest-quality “gold standard” (GS) set of spindles of MODA. This GS spindle annotation dataset introduced here is freely available on the Open Science Framework^38^ and can serve as development and testing database for automated spindle detectors including machine learning methods to analyze EEG signals. We also evaluated the performance of seven previously published spindle detectors^6,34,39–43^ against our MODA GS, breaking down performance by age and sex, and thereby providing independent benchmarking (since none of these detectors have been optimized on the MODA GS) for sleep science’s most common used spindle detectors.

## Results

### Spindle dataset collection

**Fig. 1:**
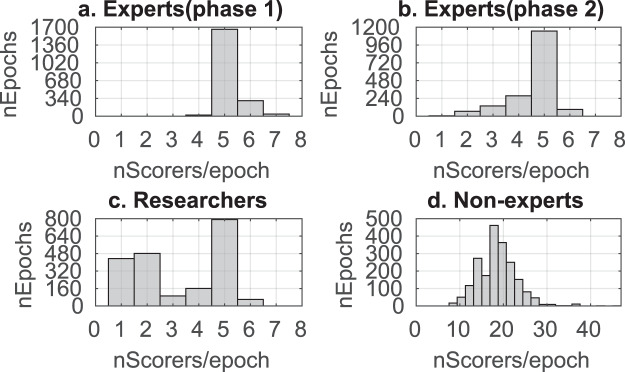
The histogram of the number of scorer views per 25 s epoch image. (**a**) PSG technologists (Experts) who viewed phase 1 (47 technologies, 10,453 epochs). (**b**) Experts who viewed phase 2 (31 technologists, 7,941 epochs). (**c**) Researchers who viewed phase 1 (18 researchers, 6,636 epochs) (**d**) Non-experts who viewed phase 1 (695 non-experts, 37,467 epochs).

Polysomnographic data from 180 subjects was sourced from the Montreal Archive of Sleep Studies (MASS)^37^. The dataset was split into two “phases”, where phase 1 consisted of 100 younger subjects (mean age of 24.1 years old) and phase 2 consisted of 80 older subjects (mean age of 62.0 years old). A subset of N2 stage sleep from the C3 channel was sampled from each subject (see methods for details). 25 sec epochs of this single channel EEG were presented to expert PSG technologists, researchers, and non-expert scorers via a custom web based scoring platform. Users identified the start and stop of candidate spindles, and indicated their confidence (high, med, low) for each spindle marked. In total, 47 PSG technologists, 18 researchers and 695 non-experts viewed 10,453, 6,636 and 37,467 epochs respectively in Phase 1. Phase 2 was viewed by 31 PSG technologists (7,941 epochs viewed). No scorers viewed the whole dataset, and the histogram of the number of scorer views per epoch image is shown in Fig. 1. A minimum number of scorers per epoch was crucial to compile a reliable gold standard (GS): the median number of scorers per epoch is 5 for the PSG technologists (Fig. 1a,b), 4 for researchers (Fig. 1c) and 18 for non-experts (Fig. 1d**)**. More than 95% of all the epochs have been seen by at least 3 PSG technologists. Table 1 presents the number of scorers and amount of data scored for each user subtype and phase. Almost 100,000 candidate spindles were identified by all scorers combined.

**Table 1:** Number of scorers and data scored for each user subtype and phase.

User subtype	PSG technologists		Researchers	Non-Experts
Phase	Phase 1 (younger)	Phase 2 (older)	Phase 1 (younger)	Phase 1 (younger)
nEpochs/scorer	10 (4–1600)	193 (20–1169)	99 (1–2018)	11 (1–2020)
nSpindles/scorer	26 (5–6438)	181 (16–1839)	137 (4–2316)	18 (0–3579)
nScorers/epoch	5 (4–7)	5 (1–6)	4 (1–6)	18 (8–47)
% epochs seen >2	100	95.8	54.7	100
total scorers	47	31	18	695
total spindles	17367	9910	7613	63935
total epochs viewed	10453	7941	6636	37467

Median number of epochs and spindles per scorer (min and max values), the median number of scorers per epoch (min and max values), the percentage of epochs seen more than twice, the total number of scorers, spindles and epochs viewed.

### Human group consensus

**Fig. 2:**
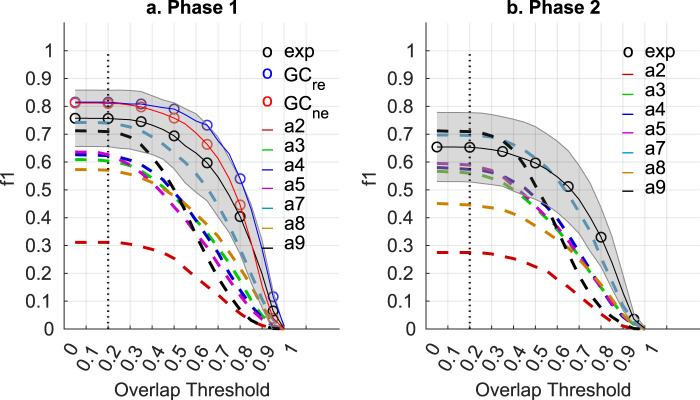
The f1-score-by-event (*f1*) in function of the overlap threshold. The average individual expert *f1* (*exp*) is shown with the ‘o’ black marker and the shaded area which is the standard deviation across experts. The Group Consensus (*GC*) of researchers (*GC*_*re*_) and non-experts (*GC*_*ne*_) are shown with the ‘o’ blue and red marker respectively. The *f1* of the automated detectors (a2-a9) are shown with dash lines. The performance is evaluated against the GS, the leave-one-out GS is used for the individual experts. (**a**) The phase 1 (younger cohort of 100 subjects). (**b**) The phase2 (older cohort of 80 subjects). An overlap threshold of 0.2 (dotted vertical line) is used for this study, as it is the strictest threshold that does not decrease performance of the detectors. As the threshold increases (i.e. spindle events must overlap with a higher percentage of the overall length of a gold standard event in order to be a match), the overall performance (*f1*) decreases.

The collected scores include many candidate spindles, and some of them showed low agreement across scorers (an event scored as a spindle by some can be scored as “not a spindle” by others). To create our GS (dataset of the highest quality spindles from the Group Consensus (GC) of experts) we averaged scoring across experts, and kept (by thresholding) only the candidate spindles that exceed a desired minimum consensus between experts – termed Group Consensus Threshold (see Methods). The minimum consensus defined by the Group Consensus Threshold (*GCt*) was chosen to maximize the mean individual expert performance (see Supplementary Fig. 1 and Table 1) against the leave-one-out *GS* (the *GS* in which the evaluated expert did not contribute to the spindle scoring). We identified an optimum required consensus *GCt* between experts of 0.2 in phase 1 and 0.35 in phase 2. These *GCts* are similar to what has been previously reported^14^. The scorers’ performance was evaluated using a “by-event” f1 score (*f1*), which is the harmonic mean between the precision and the recall. Recall is the percentage of gold standard spindles correctly detected by a scorer (true positives divided by true positives plus false negatives i.e. completeness), whereas precision is the percentage of a scorer’s spindles that are part of the gold standard set (true positives divided by false positives + true positives i.e. exactness). This by-event performance depends on how similar the estimated spindle (marked by a scorer or detected by an algorithm) has to be to the *GS* spindle to be considered as a match (True Positive); the lowest similitude occurs when spindles are adjacent (no overlap between spindles) and the strictest similitude occurs when spindles are temporally aligned with the exact same length (100% overlap). Figure 2 presents the by-event performance of experts (as well as researchers, non-experts and algorithms) as a function of the overlap threshold between estimated and *GS* spindles. An overlap threshold of 0.2 (also previously reported^14^) was the highest threshold that maximized performance and was used for further analyses in the current study.

With the GC threshold and overlap threshold chosen, the gold standard consists of 5342 spindles (3338 in phase 1, 2004 in phase 2). The properties of these spindles are reported in Table 2. This set of GS annotations is freely available on the Open Science Framework^38^, and the corresponding EEG data can be downloaded from the Montreal Archive of Sleep Studies website (http://www.ceams-carsm.ca/mass/). See the Readme document on the Open Science Framework^38^ for details on how to obtain a license to download these data.

**Table 2:** Spindle characteristics by-subject in the Gold Standard (GS) for younger (phase 1), older (phase 2) and male and females separately.

Feature	Younger	Older	Female	Male
Density (spm)	4.2 (2.4)	2.6 (2.2)	4.2 (2.4)	2.8 (2.3)
Duration (s)	0.79 (0.14)	0.75 (0.16)	0.79 (0.14)	0.76 (0.16)
Amplitude (µV)	31 (7)	26 (7)	32 (8)	27 (6)

The median (and standard deviation) spindle density, duration and amplitude across subjects are reported.

### Performance of the human group consensus and automated detectors

A rigorous evaluation of spindle results from clinical and academic sleep studies hinges on quantifying the accuracy and biases of the spindle detection method used. Therefore, to inform future work, we evaluate the spindle detection performance of experts, researchers and non-experts. Human detection of spindles is still considered the highest standard; however, many recent publications have utilized automated methods to save time and cost. Therefore, along with evaluating the performance of humans, seven popular and previously published spindle detection algorithms^6,34,39–43^ were run on the EEG data (see Methods for details on the algorithms). We compared the by-event performance of each automated detector or human group consensus (*GC*_*re*_ and *GC*_*ne*_) against the *GS*, and the individual experts were evaluated against the leave-one-out *GS* to avoid reporting bias.

**Fig. 3:**
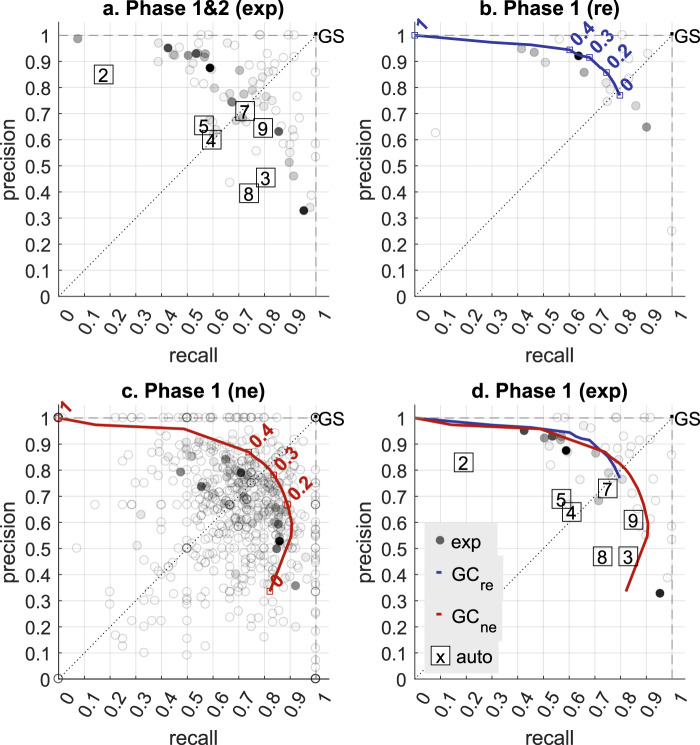
Precision-Recall plot of by-event performance of individual scorers, Groups Consensus (*GC*) and detectors. Each black ‘o’ marker represents a scorer; the intensity of the color is scaled according to how many epochs each scorer viewed. Automated detectors are labelled from 2 to 9 for a2-a9. The overlap threshold used is 0.2. The Group Consensus Threshold (*GCt*) used to create the Gold Standard (*GS*) is 0.2 for phase 1 and 0.35 for phase 2. The performance is evaluated against the GS, except for the individual experts (*exp*) which are evaluated against the leave-one-out *GS*. (**a**) The performance of the *exp* and automated detectors. Phase 1 and 2 (180 subjects) are used. (**b**) The performance of the individual researchers (*re*) and the *GC* of *re* (*GC*_*re*_) (shown in blue line). *GC*_*re*_ is created with a varying GCt from 0 to 1. The younger cohort is used (phase 1, 100 subjects). (**c**) The performance of the individual non-experts (*ne*) and the *GC* of *ne* (*GC*_*ne*_) (shown in red line). *GC*_*ne*_ is created with a varying *GCt* from 0 to 1. The younger cohort is used (phase 1, 100 subjects). (**d**) The performance of individual *exp*, automated detectors, *GC*_*re*_ and *GC*_*ne*_. The younger cohort is used (phase 1, 100 subjects).

The mean individual expert *f1* was higher in phase 1 (0.76) than phase 2 (0.65), suggesting that spindles are easier to score in the younger cohort. A mean individual expert *f1* of 0.67 has previously been reported^14^ for a cohort similar to our phase 2. The *f1* of the *GC*_*re*_ and *GC*_*ne*_ was ~0.8, suggesting that the group consensus performs better than individual experts, on average (Figs. 2a, 3d). It is noteworthy that individuals (including individual experts, non-experts and researchers) that have very high or low f1 scores tend to be scorers that did not score much data (indicated by lighter colored markers in Fig. 3). Scoring a small amount of data and thereby not encountering the full variety of epochs could have resulted in artificially high/low individual scores.

Similar to human scores, the *f*1 of the detectors were slightly reduced in the older cohort compared to the younger cohort, except for a9^43^ which remained the same (Fig. 2a,b and Supplementary Table 2). Top performance (based on *f1* score) on the younger cohort (phase 1) was the *GC*_*re*_ followed closely by the *GC*_*ne*_. The a7^42^ detector had the highest *f1* in the younger cohort, closely matching performance of the average human expert (Figs. 2a, 3d). The highest *f1* in the older cohort was reached by a9. Interestingly, a9 was the method most sensitive to the overlap threshold, as its performance decreases more rapidly than other methods as the threshold becomes more stringent (see methods). Therefore, spindles detected by the a9 algorithm and matching *GS* spindles are less perfectly temporally aligned (i.e. the start/stop and duration of spindles is less accurate) compared to the other methods. Detector a9 performance was followed closely by a7. We also evaluated the detectors performance against the *GC*_*re*_ (see Supplementary Fig. 2a) or the *GC*_*ne*_ (see Supplementary Fig. 2b). The performance of the automated methods remained essentially the same (for more details see Supplementary Table 3).

Automated detectors had their own specific tradeoff between precision (how many detected spindles were matching GS spindles) and recall (how many GS spindles were detected), the most balanced algorithms were a4 and a7 (Figs. 3a,d and Supplementary Table 2). The highest *f1* on the whole cohort (phase 1 & 2, 180 subjects) was reached by a7 (0.72 against the *GS*) which is the same as the average individual expert *f1*. This performance is followed closely by a9 with a *f1* = 0.71, a9 showed a higher recall (0.8) but a lower precision (0.65) (Fig. 3d). Figure 3(b,c) shows the Precision-Recall plot of the individual *re* or *ne* and their *GC* (*GC*_*re*_ and *GC*_*ne*_ respectively). Note that the majority of the individual researchers showed a high precision to the detriment of the recall (i.e. are overly conservative when marking spindles), and the resulting *GC*_*re*_ is perfectly balanced with a *GCt* = 0. The performance evaluation of the detectors against the three different human references (*GS*, *GC*_*re*_, *GC*_*ne*_) provided similar results (for more information see Supplementary Table 3). The number of spindles, and detailed performance metrics (True positives, False positives, False Negatives) for the *GS*, *GC*_*re*_, *GC*_*ne*_ and each automated algorithm are reported in Supplementary Table 4. The performance (as quantified by the precision, recall and f1-score) of the seven tested detectors were essentially the same as reported previously^14,34,42,43^. Note that the performance of a9 was slightly more balanced in the original publication^43^ than in the current study.

### Spindle characteristics by-subject as a function of age and sex

**Fig. 4:**
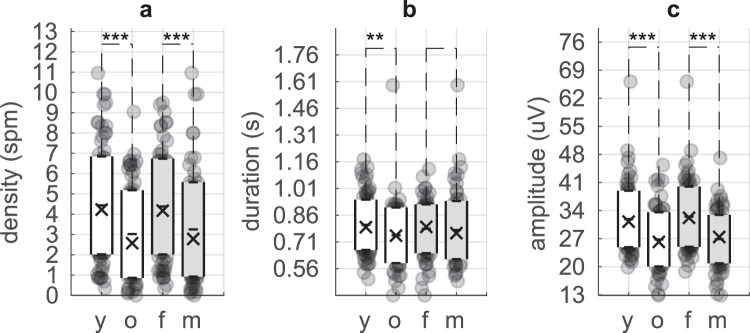
Spindle characteristics-by-subject of the Gold Standard (*GS*) as a function of age and sex. (**a**) Spindle density (spm), (**b**) Spindle duration (s) and (**c**) Spindle amplitude (µV) for younger (y; phase 1) and older (o: phase 2) subjects, females (f) and males (m). Each dot in the plot represents one subject; darker points indicate multiple subjects at the same position. The ‘−‘ marker is the mean, the ‘X’ marker is the median across subjects, and the white box shows the mean + −1 × standard deviation. The *markers show significant difference with the Mann-Whitney test: “*”p values < = 0.05, “**”p value < = 0.01, and “***”p value < = 0.001.

Spindle activity decreases with age, and sex differences have also been reported^3–13^. We evaluated the age group difference between 100 subjects 18–35 years old and 80 subjects 50–76 years old, and sex difference between the 88 females and 92 males. We tested the spindle density measured as spindle per minute (*spm*), average maximum peak-to-peak amplitude (µV), average duration (s) and average dominant oscillation frequency (Hz) by-subject on the spindle dataset included in the GS (see Methods). A 2 × 2 ANOVA showed main effect for age and sex but no interaction on both for spindle density (age p = 0.0001 and sex p = 0.001) and average amplitude (age p = 1.5e-6 and sex p = 3e-8). The difference on the average spindle duration was significant only for age (p = 0.01). No significant effect was found for the dominant oscillation frequency of the spindle. Further analyses of the age and sex differences were performed with the non-parametric Mann-Whitney test (Fig. 4) since the spindle characteristics distributions were not all normally distributed based on the Shapiro-Wilk test. The spindle density in the *GS* was higher (p = 0.0002), average duration was longer (p = 0.008) and average amplitude was higher (p = 2e-06) in younger compared to older subjects (Fig. 4). The spindle density (p = 0.0008) and the average spindle amplitude (p = 1e-06) in the *GS* were also higher in females compared to males (Fig. 4). Supplementary Tables 2 and 3 contain detailed analysis of each detector’s ability to capture the sex and age trends present in the GS.

The average spindle activity reported in the previous crowdsourcing project^14^ was similar to our phase 2 (older cohort) despite a relatively high standard deviation across subjects. Warby *et al*.^14^ reported 2.3 ± 2 spm with an average duration of 0.75 ± 0.27 s, a maximum peak-to-peak amplitude of 27 ± 11 μV and an oscillation frequency mean of 13.3 ± 1 Hz. We measured a by-subject dominant oscillation frequency of 13.1 ± 0.8 Hz (see Supplementary Table 5).

### Comparison of detection methods

When considering which method to use to detect spindles, automated or otherwise, it is important to understand which spindle properties are best captured by each. To this end, we computed the correlation of the spindle density and spindle characteristics between the *GS* spindles and automatically detected spindles for each algorithm (*a*2*-a9*) as well as *GC*_*re*_ and *GC*_*ne*_. The correlations for the spindle density in phase 1 (younger cohort, 100 subjects) are reported in Table 3. For phase 1, the correlation is higher for human GC than automated detectors. The *GC*_*ne*_ is slightly more correlated (r^2^ = 0.91) than the *GC*_*re*_ (r^2^ = 0.88). The correlation for the detectors is low for the spindle density (r^2^ average across detectors is 0.37) and spindle duration (r^2^ = 0.32), but very high for spindle amplitude (r^2^ = 0.90) and high for spindle frequency (r^2^ = 0.69). The detectors a7 and a9 performed better than the average of the detectors, especially for the spindle density which their r^2^ were 0.73 and 0.85 respectively. The correlation coefficients for the detectors in phase 2 are reported in the Supplementary Table 6. Briefly, the correlation was higher for the spindle density but lower for all the other characteristics compared to the phase 1. Again, the detectors a7 and a9 outperformed the other detectors for the correlation with the *GS* spindle density with a *r*^*2*^ = 0.83 and 0.88 respectively.

**Table 3:** Correlation coefficient r² between Gold Standard from experts (*GS*_*exp*_) and automated detectors (*a2-a9*) or group consensus of researchers (*GC*_*re*_) or non-experts (*GC*_*ne*_) for the spindle density, average duration and amplitude by-subject.

	a2	a3	a4	a5	a7	a8	a9	Auto avg	GC_re_	GC_ne_
Density spm	0.22	0.31	0.18	0.26	0.73	0.01	0.85	0.37	0.88	0.91
Duration s	0.32	0.31	0.15	0.14	0.45	0.32	0.55	0.32	0.59	0.73
Amplitude µV	0.83	0.90	0.90	0.90	0.92	0.89	0.95	0.90	0.90	0.95
Frequency Hz	0.53	0.69	0.71	0.71	0.7	0.67	0.8	0.69	0.78	0.87

The mean r^2^ across detectors is also reported (Auto avg). The correlation coefficient p-value was significant (<0.05) for each detector or human scoring except for the spindle density of a8. Only the phase 1 is shown (younger 100 subjects).

**Fig. 5:**
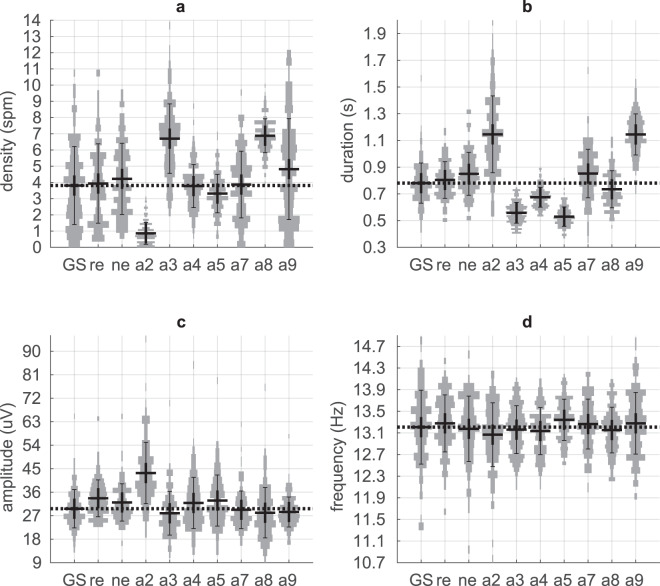
By-subject spindle characteristic distributions of the gold standard (*GS*), group consensus of researchers (*re*) group consensus of non-experts (*ne*), and each detector (*a2-a9*). Each panel refers to a different metric: (**a**) spindle density, (**b**) average spindle duration, (**c**) average spindle amplitude, (**d**) average spindle dominant frequency. The dotted horizontal line shows the average of the *GS* distribution for reference. The whole cohort is used (phase 1 & 2, 180 subjects) except for the *re* and *ne* where only the younger cohort was scored (phase 1, 100 subjects).

We compared the spindle characteristics by-subject distribution of each detector (a2-a9) and human group consensus (*GC*_*re*_ and *GC*_*ne*_) to the *GS* for the whole cohort except for *GC*_*re*_ and *GC*_*ne*_ using a Mann-Whitney test. The variance in spindle characteristics was much larger across detectors than across the three human subtypes (PSG technologists, researchers and non-experts) (Fig. 5 and Supplementary Table 7). The spindle density of a2 was much lower (0.9 spm, p = 9e-38) than the GS (3.8 spm), a3 (7 spm, p = 3.6e-25) and a8 (6.9 spm, p = 2.3e-34) were much higher than the GS. The average duration was much higher for a2 (1.15 s, p = 1.6e-33) and a9 (1.15 s, p = 2e-49) compared to the GS (0.78 s), but a3 (0.56 s, p = 4.7e-43), a4 (0.67 s, p = 1.1e-15) and a5 (0.5 s, p = 1.2e-48) were much lower. The average amplitude and oscillation frequency were about the same for all the detectors except a2 which showed spindles with greater amplitude (43 µV, p = 9.5e-30) than the GS (30 µV). The histogram at the cohort level (by-subject analysis) of the dominant oscillation frequency of spindles of the GS spindles or any of the automated detectors is unimodal, and does not support the hypothesis of decomposing the spindles into fast and slow spindles (Fig. 5d**)**. Note that the slightly higher spindle density, duration and amplitude for the *re* and *ne* spindle dataset (Fig. 5) are biased due to the fact that only the younger cohort (phase 1) was scored by these groups (see Table 2 for the true comparison for the phase 1, “Phase 1 - Younger” column).

### How many scorers are needed for crowdsourcing sleep spindle annotations?

**Fig. 6:**
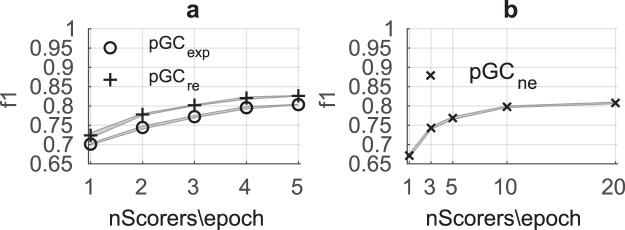
The human group consensus (GC) f1-score-by-event (*f1*) as a function of the number of scorers. (**a**) Partial group consensus of experts (*pGC*_*exp*_), each based on a different number of scorers, shown by the ‘o’ marker are evaluated against the GC of the researchers (*GC*_*re*_). Partial group consensus of researchers (*pGC*_*re*_) shown by the ‘+’ marker are evaluated against the Gold Standard made from all the experts (*GS*). (**b**) Partial group consensus of non-experts (*pGC*_*ne*_) shown by the ‘x’ marker are evaluated against the *GS*. Only the phase 1 (100 younger subjects) is used. The shaded area represents the standard deviation across the three random selections of scorers per epoch. The overlap threshold used to evaluate the performance is 0.2. Group Consensus Threshold (*GCt*) used depended of the number of scorers per epoch and the user subtypes (from 0.4 for one scorer/epoch to the optimum *GCt* for each user subtype).

Obtaining quality spindle scoring is costly and time consuming; knowing the number of scorers per epoch to achieve reliable results is worthwhile and may help to create future GS datasets. We identified that aggregating the scoring from two to four experts or researchers per epoch is optimum (Fig. 6a). However, three to ten non-experts were needed for similar performance (Fig. 6b). Zhao *et al*.^35^ reported the need for at least six non-experts to score spindles in N2 sleep stage, but the plateau of the non-experts group consensus performance (f1 < 0.8) was reached around 10 non-experts. Figure 6 shows the f1-score-by-event of five “partial” GCs, each based on different number of scorers. We evaluated these partial GC’s against the GC from another user subtype to avoid positive reporting bias. Using the leave-one-out GS was not sufficient since only few epochs include more than five experts per epoch. Therefore, partial GCs of experts (*pGC*_*exp*_) were evaluated against the GC made from the scoring of all the researchers (*GC*_*re*_), and partial GCs of researchers (*pGC*_*re*_) and non-experts (*pGC*_*ne*_) were evaluated against the formal *GS* made from the scoring of all the experts. Three random selections of the scorers per epoch were performed to see the inter-scorers/inter-epochs variation shown as a gray area. The Group Consensus Thresholds (GCt) used depended of the number of scorers per epoch and the user subtypes, from 0.4 for one scorer/epoch to the optimum *GCt* for each user subtype.

## Discussion

In this study, we describe the use of the MODA platform and crowdsourcing to generate the group consensus of a large number of human scorers for sleep spindle detection in EEG data. The group consensus of human PSG technologists (experts) is used to form the gold standard (GS), and we outline a method to evaluate the performance of the different groups of scorers, including previously reported spindle detection algorithms. The group consensus of experts and non-experts produced a high-quality spindle dataset, and the automated detectors performed, on average, worse than human scorers. Our current study (specifically phase 2, 80 older subjects from MASS^37^) is consistent with the results from our first crowdsourcing project^14^ (110 old subjects from Wisconsin Sleep Cohort^44^). The lower performance of spindle detection algorithms does not appear to be due to the age of the sleeping subjects, as we initially hypothesized^14^, as the finding is now similarly reproduced in a group of younger adults (phase 1 of MODA). The current study additionally included evaluation against a Group Consensus (GC) made from researchers scoring, and the analysis of spindle activity as a function of age and sex. Furthermore, two additional spindle detectors tested (a7^42^ and a9^43^) yielded performance equivalent to an average individual expert. To our knowledge, the MODA dataset is now the largest and most comprehensively scored sleep spindles GS available for validation of spindle detection algorithms.

The average spindle activity (such as the density, duration and amplitude) of the MODA GS for the phase 2 (80 old subjects from MASS^37^) were surprisingly consistent with the expert GS from the previous crowdsourcing project^14^ suggesting a high agreement between experts in an older cohort even across datasets. This agreement was also observed between the experts, *re* and *ne* (phase 1, 100 subjects) in the younger cohort of the current study. The high validity of our scoring allowed us to conclude the average spindle density for a young cohort was 4.2 spm with an average duration of 0.8 s, average maximum peak-to-peak amplitude of 33 µV and average dominant oscillation frequency of 13.3 Hz (activity when considering all the scorers of MODA, across all 100 subjects). The aggregated average spindle activity for older sleepers was 2.5 spm with an average duration of 0.75 s, average amplitude of 27 µV and average frequency of 13.2 Hz (MODA phase 2, 80 subjects 50–76 years old, and Warby *et al*.^14^ 110 subjects 42–72 years old). The agreement for the average spindle activity between automated algorithms was poorer than the human scoring. Only the a7 detector showed similar descriptive statistics to human scorers; i.e. average density of 3.9 spm, duration of 0.85 s, amplitude of 29 µV and frequency of 13.26 Hz for the whole cohort (phase 1 & 2). Spindles detected by a9 showed similarities with the GS spindles but the average duration was significantly longer (1.15 s). One caveat of the algorithmic performance evaluation is that the detectors were not tuned for the current dataset (instead using the default parameters suggested in their original publications). While many researchers do not tune these algorithms, the performance with tuning is potentially higher than reported here. We did not differentiate slow and fast spindles in our analysis because the oscillation frequency histogram of the spindles at the group level is clearly unimodal for the *GS*, *GCre*, *GCne* and each automated detector. The existence of slow and fast spindles could have been more obvious in our database with the analysis of additional channels, such as a frontal channel for slow spindles and a parietal channel for fast spindles^6,45,46^.

Most of the detectors tested in our study showed the same significant age and sex differences as the experts, which, in-turn, matches the literature^3–13^. However, algorithms a7 and a9 detected an additional significant sex difference: the spindles were on average longer in females, a finding which until now has only been seen at a trending significance level (p0.05 < p < 0.1)^4,7^. We did not detect this effect in our own GS (only a trend in correct direction was observed, with p = 0.2), and this potentially points to a7 and a9 detectors being more discriminatory than human scoring. The a8^34^ detector was alone in showing an opposite age and sex effect for the spindle density. Not all detectors performed equally: the correlation of the by-subject spindle density between the GS and the detectors was generally low (an average r^2^ across detectors of 0.37) compared to the human group consensus (*GCre* r^2^ = 0.88 and *GCne* r^2^ = 0.91) in the younger cohort, however the detectors a7 (r^2^ = 0.73) and a9 (r^2^ = 0.85) performed well. Algorithm performance was slightly better in the older cohort (average r^2^ across detectors = 0.47), and again a7 (r^2^ = 0.83) and a9 (r^2^ = 0.88) performed well. Spindle density and amplitude was more accurately captured compared to spindle duration: correlations between GS and detectors/humans were generally lower for duration than for density (even for the human Group Consensus, *GC*_*re*_ r^2^ = 0.59 and *GC*_*ne*_ r^2^ = 0.73), and all detectors and human GC had a high correlation with the GS for the average spindle maximum peak-to-peak amplitude.

Creating an optimal GS is central to maximizing dataset validity. Obtaining the highest number of scorers with the highest level of expertise possible is, of course, the best scenario to create this optimal GS. However, our study suggests that collecting the scores of three researchers (*re*) or 10 non-experts (*ne*) provided a GC *f1* of 0.8 against the expert’s GS, providing a performance similar to the average individual expert (*f1* = 0.76) (only observed in phase 1 since the phase 2 was not scored by *re* or *ne*). Comparing the spindle detectors to the GC of *re* (*GC*_*re*_) or *ne* (*GC*_*ne*_) allowed the same conclusion about their performance as the experts’ comparison. The *GC*_*re*_ proved to be a valid standard reference despite the high precision and the moderate recall of the researchers. Creating a GC where the f1-score is maximized effectively forced the GC to be balanced between the recall and the precision. We also identified that aggregating the scoring from two to four experts/researchers or three to ten non-experts is sufficient, and after this point, the performance of the GC begins to plateau.

Throughout the analysis, we have used a relaxed overlap threshold (only 20% overlap between a potential detection and a spindle in the GS was required to be a true positive). Clearly a higher threshold is desirable in practice, but we wanted to present the best performance possible for the automated detectors. All automated detectors decay in performance with increasing overlap threshold faster than human scorers, meaning that automated detectors do not predict the start/stop and duration of spindles similarly to humans. Using a stricter threshold such as 80% would produce an even larger difference between human and automated scoring performance. In this regard, the a9 detector, which had some of the highest performance scores with an overlap of 20%, was unique in that it had the most rapid decline of performance with increasing overlap threshold requirements (Fig. 2). The preference of the a9 detector to find very long spindles may be an area of potential improvement for this particular algorithm.

It should be clearly noted that the use of human-scored spindles as the gold standard is open for debate. In our study, the scoring performance reported and descriptive statistics of the MODA GS spindles are “true” only in the sense that many human experts agree on them. The lesser performance of spindle algorithms is only relative to human scoring. It remains unclear whether the algorithmically detected “hidden spindles” that are missed by humans are mechanistically identical to human detected spindles, spurious, or perhaps separate and biologically meaningful phenomena. Individual spindle detection algorithms may prove to be superior for specific uses, such as disease biomarkers, markers of cognition and intelligence, or in cases of co-recorded EEG and fMRI, where the signal-to-noise ratio becomes more challenging. What remains clear however, is that individual spindle detection algorithms find different sets of spindles relative to human scoring, and different than other spindle algorithms. Since the different algorithms are not entirely consistent with each other, it is difficult to use any one detector as the gold standard. Therefore, if you are designing an automated detector to match human scoring, then validation against the MODA GS is the best choice.

The variance in automated detectors means choosing one is not a one-size-fits-all process. Some detectors may be better in characterizing the spindle activity of unhealthy subjects, subjects under different conditions or to reveal specific features of spindles. For example, out of the seven detectors tested in the current study a2^39^ was the best in separating Parkinson’s disease patients from controls (unpublished results conducted in our lab). Furthermore, a8 showed poor results in the current study, but performed well when compared to an expert (f1 = 0.71) or a group consensus of non-experts (f1 = 0.73) who scored on a band-pass filtered 11–16 Hz EEG signal^34^. The a7 detector was the most similar to the human scoring, which is not surprising considering it was designed to emulate expert human scoring, and it has been trained on a human GS^42^. The detector a9 showed high performances in the current study. It is based on a non-linear model to separate the transients from the sustained rhythmic oscillations of the spindle^43^. The detector a9 is also proposed to pre-process the raw EEG prior to the spindle detection (possibly combined to another automated detector)^47^, a design possibly of interest for noisy EEG signals such as those recorded in fMRI. Instead of choosing the top performing algorithm defined here (e.g. a7), researchers might consider testing multiple or even a combination of detectors. For example, testing multiple published detectors initially (ideally on pilot data) to establish which detector is the most useful for their application, they could then use that method consistently for all future work, thereby allowing valid comparison between versions of their work. Specific research areas may focus on specific properties of the spindle signal (e.g. require amplitude sensitivity rather than frequency sensitivity), and, as shown here, some detectors are more sensitive specific signal properties. Therefore, automated algorithms to detect spindles may also be chosen based on the specific field of inquiry and their history in answering specific research questions. Overall, choosing appropriate spindle detection requires efforts from the researchers to standardize the evaluation of the detectors. A common set of spindles to compare with, e.g. the MODA GS, is one important step of this standardization.

There are some limitations to the current work. Producing a higher quality GS might be achieved with more experts (although see our recommendations for a sufficient number of scorers), but also by improvements to the MODA web interface. An interface which better replicates the PSG technologist work environment, such as presenting a complete montage of channels, the possibility to go back and forth between epochs, and displaying a whole night per subject, and may yield higher validity expert annotations. Furthermore, the current GS includes only healthy subjects from 18–76 years old (distributed non-uniformly), and focuses on spindles in stage 2 and the C3 channel; different GS could be created from other populations, channels or stages.

With the release of the MODA annotations dataset, we hope to spur development of reliable, generalizable automatic sleep analysis tools. Complex models with many parameters (such as those in machine learning) are prone to overfitting (i.e. fitting dataset specific noise), and therefore, the reported accuracy of detectors may be inflated, and results may not generalize to new, unseen data. We suggest that developers should train, validate and test their algorithm with a nested cross-validation on the MODA GS.

In conclusion, our study demonstrates that crowdsourcing with experts, researchers and non-experts replicates well, and is a viable method for generating a large dataset of EEG events. We trust that the MODA interface and the GS dataset generated from it will prove a popular tool for researchers to collect data, train and validate automated detectors, and act as a standardized benchmark for selecting the most appropriate algorithm for specific research goals. The MODA dataset was a concerted effort, and highlights the importance of open, transparent and collaborative research. In this vein, we encourage all developed algorithms to be open source so that these tools may help us understand sleep further, including how spindles play a role in memory and mental disorders.

## Methods

### EEG data

Polysomnographic data used came from the Montreal Archive of Sleep Studies (MASS)^37^; 180 subjects were sampled from the SS1-SS5 subsets. The dataset was split into “phase 1” and “phase 2”; 100 younger subjects (mean age of 24.1 years old) and 80 older subjects (mean age of 62.0 years old) respectively. “Blocks” of 115 s were randomly extracted from artifact free Stage N2 sleep. Three blocks (~6 mins) were extracted in 85 subjects in phase 1 and 65 subjects in phase 2; and 10 blocks (~20 mins) were extracted in the remaining 15 subjects of each phase. Almost 24 h of EEG time series was extracted to be scored. Table 4 presents the demographic information of subjects sampled and the amount of EEG data extracted. C3 channel was reformatted to C3-A2 when possible otherwise the original reference “C3-Linked Ear” (*C3-LE*) was kept. We band-pass filtered the signal between 0.3–30 Hz as suggested by AASM^22^ and down sampled it to 100 Hz to reduce processing time.

**Table 4:** Data collection to score spindles.

Phase	Subjects (ratio female)	Mean Age years (min-max)	Number of epochs	Total duration (h)
1	100 (0.52f)	24.1 (18–35)	2020	12.9
2	80 (0.46f)	62.0 (50–76)	1725	11
Total	180 (0.49f)	41.0 (18–76)	3745	23.9

Number of subjects selected from MASS^37^ and the number of epochs extracted with the corresponding amount of time (h) for each phase.

### Signal processing

Band-pass filter 0.3–30 Hz is implemented in MATLAB 2016b (MathWorks, Inc., Natick, MA, USA). The filter characteristics are Butterworth IIR 10th order. The filter is constructed with zero-pole-gain form converted into a Second Order Section (SOS) and the non-linear phase is corrected by the “filtfilt” function. The EEG down sampling to 100 Hz is also implemented in MATLAB 2016b (MathWorks, Inc., Natick, MA, USA) with the function “resample” which has been called to use a polyphase antialiasing filter.

### MODA Web interface developed to collect spindle scoring

**Fig. 7:**
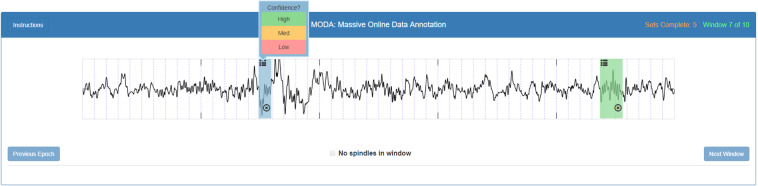
An example of 25 s epoch image to score spindles with confidence intervals on the MODA (Massive Online Data Annotation) website.

We developed a custom JavaScript web interface, called MODA, to collect the annotations of a large number of scorers. Signals to be scored on MODA must be encoded as images, therefore the extracted data blocks of 115 s (C3 EEG channel) were converted into 5 epoch images of 25 s (overlap of 2.5 s between consecutive epochs). Images were 10” wide per 1” high in five resolutions from 80 dpi to 125 dpi to suit the most common monitors. Negative voltages (+100 µV to −100 µV) were displayed upward to present data time series in a familiar way to experts. The scorers were first asked to register and complete a simple profile about their experience in sleep scoring (if any). A short description of how the interface works, and how to score spindles, was presented. The American Academy of sleep medicine’s (AASM’s)^22^ spindle criteria were used to develop the instructions to score spindles. All the scorers underwent 10 practice trails with feedback; they were asked to draw boxes around each spindle they saw and rate the confidence (as “high”, “medium” or “low”) that each box contains a spindle (Supplementary Fig. 3**)**. After the completion of the practice session, they were allowed to score spindles (possibly in multiple short sessions) for the MODA dataset (Fig. 7). Phase 1 dataset (younger cohort) was presented first. Images were displayed as a “set” of 2 blocks (i.e. 10 epochs) to scorers. The same “set” was presented to different scorers until the desired number of views was reached. The number of sets scored was shown, but the total number of sets left to score was unknown for each scorer. Epochs may contain no spindles and there was no limit on the number of spindles that could be present.

### MODA scorers

Scorers consisted of PSG Technologists (registered as Polysomnographic Technologists on www.brpt.org/rpsgt), designed the experts (*exp*) in our study, Researchers (*re*) with experience in scoring sleep, and Non-Expert (*ne*) “MTurkers” recruited from Amazon Mechanical Turk. PSG technologists and researchers were recruited through on-line announcements, scientific conferences, word of mouth, and from the authors’ personal database.

### Creating the MODA group consensus

The visual scoring of spindles needs practice since other signal features also mimic spindles and they can be partially hidden or deformed; therefore, only spindles that have been marked with a certain agreement between scorers should be kept to form a high-quality set of spindles noted Group Consensus (GC). To increase the scoring quality, we asked to scorers to rate their confidence (low, med, high) for each spindle marked. Specifically, each sample of the EEG time series had a score weighted by the confidence rate given by the scorer; 1 for high, 0.75 for medium, 0.5 for low confidence and 0 for no spindle at all. Then, sample by sample, the scores were averaged across scorers, and if they exceed the chosen Group Consensus Threshold (*GCt*) then these samples were identified to be part of the GC spindle dataset. In this way, either some scorers must be certain, or many scorers must be moderately confident for a location to be marked as a GC spindle. The three subtypes of users who scored on MODA: “*exp*, *re* and *ne*” allowed the creation of different *GC*. The GC of experts was considered the highest-quality set of spindles of MODA, and therefore was designated as the formal “gold standard” GS of MODA. The *GCt* used to create our *GS* was chosen to maximize the average individual performance across experts. Each individual expert was evaluated against a GS which did not include its own scores (leave-one-out GS) to avoid a positive reporting bias. The *GCt* used to form the *GC* of *re* (*GCre*) or *ne* (*GCne*) were chosen to maximize the GC performance against the GS made from all the experts. These thresholds are arbitrary, and others may want to use a different aggregation method or thresholds to create their own GC. Additional clean-ups, on the created GC, were made to increase their validity. A spindle shown on two consecutive epochs (during the 2.5 s overlap of epochs) may be detected more easily on either epoch. Therefore, for the set of samples that occur on two epochs, we consider the highest score for each scorer. Too short (<0.3 s) adjacent (<0.1 s apart) spindles were merged, and spindle longer than 2.5 s or shorter than 0.3 s were filtered out of the GC.

### Performance evaluation

The performance evaluation followed the strategy described in the previous spindle crowdsourcing project^14^. The primary performance evaluation was approached ‘by-event’, meaning that spindles are considered to be variable length events. An overlap rule must therefore be applied to determine if two variable length and partially overlapping events (estimated spindle and GS spindle) can be considered a match. The recall (fraction of GS spindles found: $\frac{TP}{TP+FN}$), the precision (fraction of events that matches GS spindles: $\frac{TP}{TP+FP}$) and the f1-score $\left(2\times \frac{precision\times recall}{precision+recall}\right)$ (where *TP* is the number of True Positive, *FP* the False Positive and *FN* the False Negative) were used since spindles are relatively rare events. To consider an estimated spindle (detection) as correctly matching a GS spindle (event), the detection must overlap the event above a certain overlap threshold. The overlap is computed as the intersection (the part of event detected) over the union (sum of the length of the event and the detection) between the event and the detection. Only one detection can match an event, the one with the greatest overlap, other detections overlapping the same event are considered FP. The overlap threshold chosen was the strictest threshold that did not penalize any of the human group consensuses or automated algorithms. A low overlap threshold (0.2 was previously reported^14^) allows detections to be shorter or longer than the GS spindle or being not perfectly aligned with the GS spindle. In addition, the performance evaluation was done at the ‘by-subject’ level. The multiple detections or measurements that belong to the same individual EEG recording (sleeping night of one subject) were aggregated into a single average for that subject. These characteristics are the spindle density measured as the number of spindles per minute (spm), the average spindle duration (s), amplitude (µV) and frequency (Hz). In detail, the amplitude was computed as the maximum peak-to-peak amplitude of the spindle band-pass filtered 11–16 Hz. The frequency was computed as the dominant oscillation frequency of the spindle through FFT (Fast Fourier Transform). An FFT with five seconds zero-padding was performed on the EEG signal of the spindle band-pass filtered 10–16 Hz, and the frequency with the maximum energy was extracted. The frequency histogram at the cohort level was generated to evaluate the opportunity of breaking down spindles into fast and slow. The by-subject analysis allowed looking at the correlation of the spindle density or characteristics with the GS. The by-subject performance can be high compared to the by-event performance if the detection bias (such as recall or precision) is constant across subjects (ex. detections are consistently 0.5 s longer or delayed by 0.2 s compared to the GS spindles).

### Automated spindle detectors tested

To provide a framework of how to test automated algorithms on the MODA GS, we evaluated the performance of seven previously published spindle detectors^6,34,39–43^ (for more details about their respective design see Table 5). These detectors were selected because of the prevalence of their use, the requirement that they only need one EEG channel to perform the analysis, and the availability of open source Matlab code to facilitate their implementation. Detectors were run “out-of-the-box” with the default parameters suggested in their corresponding publications. The detectors were evaluated first by-event against the MODA GS and secondly against the GC made from the researchers (*GC*_*re*_) or non-expert scoring (*GC*_*ne*_). The by-subject analysis was also performed in order to compare their spindle density and average spindle characteristics to the human scoring. Age and sex differences for the spindle activity were also tested for each detector. Reported performances are valid “out-of-sample” performance since none of these detectors have been developed or trained on the MODA GS. Even if the EEG data for MODA comes from the open source MASS^37^ dataset, only 15 subjects (out of the 180 subjects used for MODA) have existing spindles scored (and by only 2 experts compared to an average of 5 experts per epoch in our data). Furthermore, one of the previous experts from the MASS spindle dataset did not score in the same manner as MODA (looking at the band-pass filtered signal 11–16 Hz instead of looking only at the broad-band (0.3–30 Hz) C3 channel).

**Table 5:** Simplified descriptions of spindle detector algorithms tested. See original publication for more details.

a2^39^	Band-pass filters (11–15 Hz) the EEG signal to compute its envelope. An upper (8 x mean) and lower (2 × mean) thresholds are used to detect spindles. *Code available on github.com/swarby/SpindleAlgorithms_NatMeth_2014*
a3^40^	Band-pass filters (11.3–15.7 Hz) the EEG signal to compute the root mean squared (RMS) on sliding windows (100 ms length with a step of 50 ms), and then applies a threshold (1.5 × STD). *Code available on github.com/swarby/SpindleAlgorithms_NatMeth_2014*
a4^6^	Band-pass filters (11–15 Hz) the EEG signal to compute the RMS on sliding windows (25 ms length with a step of 25 ms), and then applies a threshold (95^th^ percentile). *Code available on github.com/swarby/SpindleAlgorithms_NatMeth_2014*
a5^41^	Transforms the EEG signal into continuous Morlet wavelets to compute the moving average on sliding windows (0.1 sec length), and then applies the threshold (4.5 × mean). *Code available on github.com/swarby/SpindleAlgorithms_NatMeth_2014*
a7^42^	Computes the absolute (Mean Square) sigma (11–16 Hz) power, the relative sigma power with Power Spectral Analysis, the covariance and correlation between sigma filtered and the unfiltered EEG signal on sliding windows (0.3 sec length with a step of 0.1 sec). It then detects a spindle if the 4 features extracted from EEG exceed their respective threshold (1.25 µV^2^, 1.6 × STD, 1.3 × STD and 69%). *Code available on github.com/swarby/A7_LacourseSpindleDetector*
a8^34^	Decomposes the EEG signal into 3 components: Direct Current, oscillation around 13.5 Hz and other frequency components (0.3–30 Hz). Spindles are detected from the oscillation around 13.5 Hz with an upper (2.33 × STD) and lower (0.1 × STD) thresholds applied in sliding windows (60 sec length). *Code available on github.com/stuartfogel/detect_spindles*
a9^43^	Decomposes the EEG signal into 3 components: transient (t), low-frequency (lf) and oscillations (s). *S* are represented sparsely with Short Time Fourier Transform (1.28 sec length with a step of 0.32 sec). It then detects spindles by thresholding (c1 = 0.03) the Teager-Kaiser energy operator (energy smooth) of *s* band-pass filtered (11.5–15.5 Hz). Parameters initialization: lambda0 = 0.6, lambda1 = 7, lambda2 = 8.5, mu = 0.5 and c1 = 0.03. *Code available on github.com/aparek/detoks*

## Supplementary information

Supplemental Material

## Data Availability

The dataset generated in the current study is described on the Open Science Framework (OSF)^38^, it includes links to the spindle annotations and instructions on how to obtain the PSG data used (MASS^37^ dataset). See the wiki on the OSF site, and Readme on linked Github repository for more information on how to download the data. The PSG files can be requested as described on the MASS web page (http://www.ceams-carsm.ca/mass). Sharing occurs after the requirements of the MASS databank application are met.

## Code availability

The JavaScript code of the MODA interface developed to collect the annotations is open source^48^. The Matlab code to manage the PSG files and generate the GS from the spindle scoring files is also open source^38^.
