# Supplementary material for: Massive online data annotation, crowdsourcing to generate high quality sleep spindle annotations from EEG data
Source: Sci Data. 2020 Jun 19;7:190. doi: 10.1038/s41597-020-0533-4 (PMC7305234; doi:10.1038/s41597-020-0533-4)
Supplement: Supplementary file 1 — Supplemental Material [file 41597_2020_533_MOESM1_ESM.pdf]

# Table of contents

## Contents

Table of contents ........ 1
Supplementary Results ........ 2
Group consensus threshold vs agreement ........ 2
Optimal consensus thresholds for each scorer type and phase ........ 3
Performance as a function of overlap threshold ........ 4
Performance of automated detectors for each phase and whole cohort. ........ 5
Performance of each detector against researcher and non-expert consensus ........ 6
Number of spindles detected by each algorithm ........ 7
Age differences in spindle characteristics for each detector ........ 8
Sex differences in spindle characteristics for each detector ........ 9
Spindle dominant frequency for gold standard and each detector ........ 10
Correlation between gold standard and each detector for each spindle characteristic in phase 2 ........ 11
The average spindle density, duration, amplitude and frequency for each detection method ........ 12
An example of a practice trail in the MODA web interface ........ 13

# Supplementary Results

## Group consensus threshold vs agreement

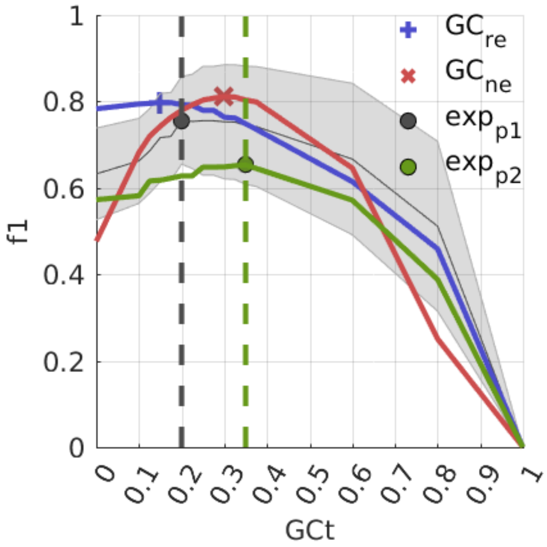


**Supplementary Figure 1.** The f1-score (*f1)* of the Group Consensus of researchers ($GC_{re}$) and non-experts ($GC_{ne}$) and the average individual expert *f1*. The *f1* of $GC_{re}$ and $GC_{ne}$ are evaluated against the GC of experts (*GS)*, and the individual expert (*exp*) is evaluated against the leave-one-out *GS* for the phase 1 (*p1*) and the phase 2 (*p2*). The shaded area represents the standard deviation (*std*) across the *f1* of the *exp*, the *std* of $exp_{p2}$ is similar and it is not shown for clarity. The x-axis shows different *GCt* applied to the scores of each user subtype.

## Optimal consensus thresholds for each scorer type and phase

| subtype users | CGt | Identification of the optimum Group Consensus Threshold |
|---|---|---|
| Experts (phase 1, younger) | 0.2 | Maximized the average individual expert f1 against the leave-one-out GS in phase 1. |
| Experts (phase 2, older) | 0.35 | Maximized the average individual expert f1 against the leave-one-out *GS* in phase 2. |
| Researchers (phase 1, younger) | 0.15 | Maximized the f1 of the GC of researchers against the *GS* in phase 1. |
| Non-experts (phase 1, younger) | 0.3 | Maximized the f1 of the GC of non-experts against the *GS* in phase 1. |
| Researchers (phase 1, younger) | 0.125 | Maximized the average individual researcher f1 against the leave-one-out GC made from the researchers scoring ($GC_{re}$). |

**Supplementary Table 1.** Optimum Group Consensus Threshold (*GCt*) for the different user subtypes and phases. The *GCt* values and how they were identified are reported.

## Performance as a function of overlap threshold

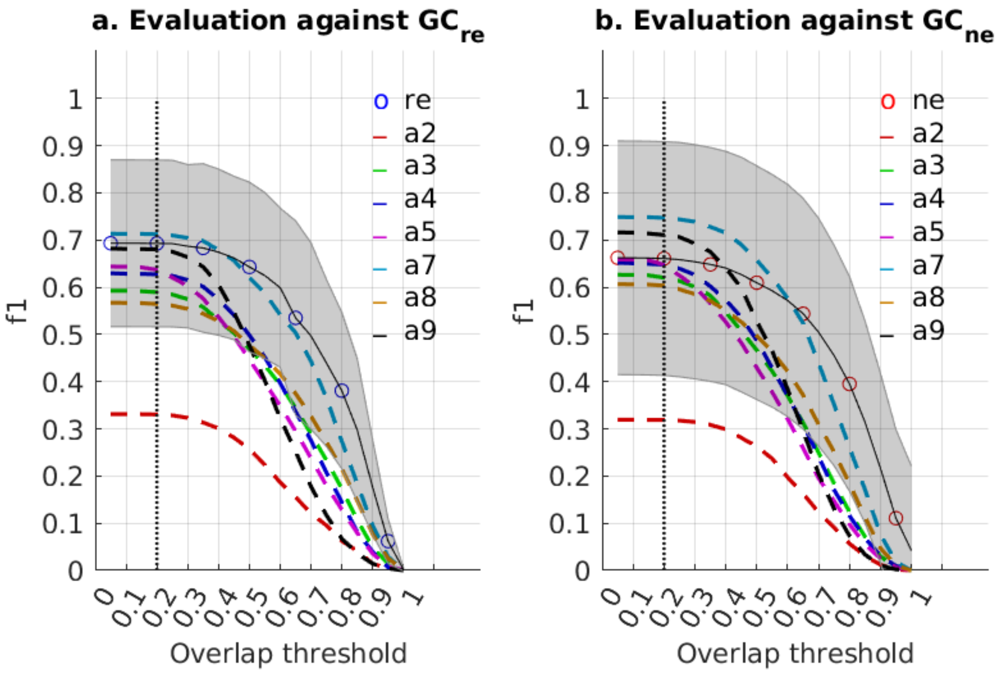


**Supplementary Figure 2.** The f1-score-by-event (*f1*) as a function of the overlap threshold. Human scorings represented with 'o' markers are the average individual *f1* and the shaded area is the standard deviation across individuals. The *f1* of the automated detectors (a2-a9) are shown with dash lines. Only the phase 1 (younger) is used (100 subjects). (a) The individual researchers (*re)* is evaluated against the leave-one-out Group Consensus of researchers ($GC_{re}$) and a2-a9 are evaluated against the $GC_{re}$. The Group Consensus Threshold (*GCt*) used is 0.125 (b) The individual *ne* and a2-a9 are evaluated against the Group Consensus of non-experts ($GC_{ne}$), and the *GCt* used is 0.3.

## Performance of automated detectors for each phase and whole cohort.

| | **(a)** Phase 1 (younger) | | | **(b)** Phase 2 (older) | | | **(c)** Whole Cohort | | |
|---|---|---|---|---|---|---|---|---|---|
| | Recall | Precision | f1 | Recall | Precision | f1 | Recall | Precision | f1 |
| a2 | 0.19 | 0.83 | 0.31 | 0.16 | 0.87 | 0.27 | 0.18 | 0.85 | 0.29 |
| a3 | 0.83 | 0.47 | 0.60 | 0.78 | 0.44 | 0.56 | 0.81 | 0.46 | 0.58 |
| a4 | 0.61 | 0.64 | 0.62 | 0.58 | 0.56 | 0.57 | 0.60 | 0.60 | 0.60 |
| a5 | 0.57 | 0.69 | 0.63 | 0.56 | 0.62 | 0.59 | 0.57 | 0.66 | 0.61 |
| a7 | 0.75 | 0.73 | 0.74 | 0.70 | 0.69 | 0.70 | 0.73 | 0.71 | 0.72 |
| a8 | 0.73 | 0.47 | 0.57 | 0.75 | 0.32 | 0.45 | 0.74 | 0.40 | 0.51 |
| a9 | 0.85 | 0.61 | 0.71 | 0.74 | 0.68 | 0.71 | 0.80 | 0.65 | 0.71 |
| IExp | 0.76 + 0.16 | 0.81 + 0.17 | 0.76 + 0.10 | 0.66 + 0.19 | 0.74 + 0.17 | 0.65 + 0.12 | 0.72 + 0.18 | 0.78 + 0.17 | 0.72 + 0.12 |

**Supplementary Table 2.** By-event performance of automated detectors (a2-a9) and individual experts (IExp) against the (*GS*). Performance is presented as recall, precision and f1-score (*f1*) for (a) the phase 1 (young cohort, 100 subjects), (b) the phase 2 (old cohort, 80 subjects) and (c) the whole cohort (phase 1 & 2). The performance of the IExp is presented as the mean (and standard deviation) across experts (47 experts for phase 1 and 31 for phase 2). The overlap threshold used is 0.2. The Group Consensus Threshold (*GCt*) used is 0.2 for phase 1 and 0.35 for phase 2.

## Performance of each detector against researcher and non-expert consensus

| | **(a)** Evaluation against $GC_{re}$ | | | **(b)** Evaluation against $GC_{ne}$ | | |
|---|---|---|---|---|---|---|
| | recall | precision | f1 | recall | precision | f1 |
| a2 | 0.21 | 0.84 | 0.33 | 0.20 | 0.86 | 0.32 |
| a3 | 0.84 | 0.45 | 0.59 | 0.84 | 0.49 | 0.62 |
| a4 | 0.63 | 0.62 | 0.63 | 0.62 | 0.67 | 0.65 |
| a5 | 0.60 | 0.68 | 0.64 | 0.59 | 0.72 | 0.65 |
| a7 | 0.75 | 0.68 | 0.71 | 0.75 | 0.74 | 0.75 |
| a8 | 0.75 | 0.45 | 0.56 | 0.76 | 0.50 | 0.60 |
| a9 | 0.85 | 0.57 | 0.68 | 0.84 | 0.62 | 0.71 |
| Ire | 0.71 + 0.22 | 0.77 + 0.17 | 0.69 + 0.18 | NA | NA | NA |
| Ine | NA | NA | NA | 0.71 + 0.27 | 0.74 + 0.22 | 0.66 + 0.25 |

**Supplementary Table 3.** By-event performance of automated detectors (a2-a9), individual researchers (*Ire*) and individual non-experts (*Ine*). Performance is presented as recall, precision and f1-score (*f1*). The performance of the individual scorers is presented as the mean (and standard deviation) across the group of scorers (18 researchers or 695 non-experts). (a) The performance evaluation is against the Group Consensus of *re* ($GC_{re}$), and the *Ire* are evaluated against the leave-one-out $GC_{re}$. The Group Consensus Threshold (*GCt*) of 0.125 is used. (b) The performance evaluation is against the Group Consensus of *ne* ($GC_{ne}$) with a *GCt*=0.3. The younger cohort (100 subjects) is used. The overlap threshold used is 0.2.

## Number of spindles detected by each algorithm

| | **(a)** Phase 1 (younger) | | | | **(b)** Phase 2 (older) | | | |
|---|---|---|---|---|---|---|---|---|
| | nSpindles | TP | FN | FP | nSpindles | TP | FN | FP |
| GS | 3338 | - | - | - | 2004 | - | - | - |
| $GC_{re}$ | 2948 | 2508 | 830 | 440 | NA | NA | NA | NA |
| $GC_{ne}$ | 3396 | 2732 | 606 | 664 | NA | NA | NA | NA |
| a2 | 768 | 640 | 2698 | 128 | 372 | 327 | 1677 | 45 |
| a3 | 5786 | 2759 | 579 | 3027 | 3580 | 1568 | 436 | 2012 |
| a4 | 3153 | 2025 | 1313 | 1128 | 2057 | 1167 | 837 | 890 |
| a5 | 2774 | 1917 | 1421 | 857 | 1805 | 1125 | 879 | 680 |
| a7 | 3426 | 2509 | 829 | 917 | 2044 | 1412 | 592 | 632 |
| a8 | 5183 | 2439 | 899 | 2744 | 4722 | 1504 | 500 | 3218 |
| a9 | 4643 | 2839 | 499 | 1804 | 2172 | 1485 | 519 | 687 |

**Supplementary Table 4.** The number of spindles in the Gold Standard (*GS*), the Group Consensus of researchers ($GC_{re}$) and non-expert ($GC_{ne}$), and detected by each algorithm (a2-a9) including the True Positive (TP), False Positive (FP) and False Negative (FN). The overlap threshold used is 0.2. (a) Phase 1 (younger, 100 subjects) is used. The Group Consensus Threshold (*GCt*) for the *GS* is 0.2, *GCt*=0.15 for $GC_{re}$ and *GCt*=0.3 for $GC_{ne}$. (b) Phase 2 (older, 80 subjects) is used. A GCt=0.35 is used for the *GS*.

## Age differences in spindle characteristics for each detector

| | Density spm | | | Duration s | | | Amplitude µV | | |
|---|---|---|---|---|---|---|---|---|---|
| Age | Younger | Older | P | Younger | Older | P | Younger | Older | P |
| GS | 4.2(2.4) | 2.6(2.2) | 1.65E-04 | 0.79(0.14) | 0.75(0.16) | 7.63E-03 | 31( 7) | 26( 7) | 1.73E-06 |
| a2 | 0.9(0.7) | 0.5(0.6) | 2.78E-07 | 1.22(0.28) | 1.00(0.23) | 3.85E-07 | 43(12) | 38(10) | 1.34E-02 |
| a3 | 7.5(1.9) | 5.6(1.8) | 3.22E-11 | 0.59(0.07) | 0.51(0.07) | 2.31E-09 | 29( 9) | 25( 7) | 8.74E-05 |
| a4 | 4.2(1.2) | 3.2(1.3) | 2.05E-07 | 0.69(0.07) | 0.65(0.07) | 1.08E-03 | 33(10) | 28( 8) | 3.25E-05 |
| a5 | 3.7(1.1) | 2.7(1.1) | 8.97E-08 | 0.54(0.06) | 0.50(0.08) | 1.47E-04 | 34(10) | 29( 9) | 3.50E-05 |
| a7 | 4.7(1.9) | 3.0(2.0) | 2.52E-06 | 0.91(0.16) | 0.76(0.17) | 5.42E-10 | 30( 7) | 26( 6) | 1.04E-04 |
| a8 | 6.6(0.9) | 7.2(1.1) | 2.49E-03 | 0.80(0.13) | 0.64(0.10) | 4.40E-14 | 30(10) | 23( 8) | 7.59E-07 |
| a9 | 6.1(3.1) | 2.8(2.4) | 6.05E-10 | 1.20(0.14) | 1.09(0.15) | 1.35E-06 | 29( 6) | 26( 6) | 1.11E-03 |

**Supplementary Table 5.** Spindle characteristics-by-subject of the Gold Standard (GS) and the automated detectors (a2-a9) for younger and older subjects. The median (and standard deviation) spindle density, duration and amplitude across subjects are reported. The p value (*P*) of the Mann-Whitney test between younger and older groups for each spindle characteristic is also reported. The whole cohort is used (phase 1 & 2, 180 subjects).

## Sex differences in spindle characteristics for each detector

| | Density spm | | | Duration s | | | Amplitude µV | | |
|---|---|---|---|---|---|---|---|---|---|
| Sex | F | M | P | F | M | P | F | M | P |
| GS | 4.2(2.4) | 2.8(2.3) | 7.68E-04 | 0.79(0.14) | 0.76(0.16) | 2.26E-01 | 32( 8) | 27( 6) | 1.16E-06 |
| a2 | 0.9(0.7) | 0.7(0.6) | 3.89E-04 | 1.11(0.25) | 1.13(0.32) | 6.26E-01 | 47(12) | 38(10) | 5.53E-06 |
| a3 | 7.1(2.0) | 6.4(2.2) | 5.74E-02 | 0.56(0.07) | 0.56(0.09) | 9.33E-01 | 31( 8) | 24( 7) | 3.68E-07 |
| a4 | 3.8(1.3) | 3.7(1.3) | 1.28E-01 | 0.68(0.08) | 0.67(0.07) | 5.17E-01 | 35(10) | 27( 8) | 4.35E-07 |
| a5 | 3.4(1.1) | 3.3(1.3) | 3.44E-01 | 0.52(0.07) | 0.52(0.07) | 7.85E-01 | 36(10) | 28( 8) | 7.49E-08 |
| a7 | 4.5(2.0) | 3.3(2.0) | 1.67E-03 | 0.90(0.15) | 0.82(0.20) | 3.49E-03 | 31( 7) | 26( 6) | 3.96E-08 |
| a8 | 6.6(1.0) | 7.1(1.1) | 2.18E-03 | 0.71(0.12) | 0.71(0.15) | 8.10E-01 | 31(10) | 24( 8) | 5.29E-07 |
| a9 | 5.6(3.1) | 3.7(3.0) | 5.83E-03 | 1.21(0.13) | 1.09(0.16) | 2.12E-05 | 31( 6) | 26( 5) | 7.75E-10 |

**Supplementary Table 6.** Spindle characteristics by-subject in the Gold Standard (GS) and the automated detector (a2-a9) for females (F) and males (M). The median (and standard deviation) spindle density, duration and amplitude across subjects are reported. The p value (P) of the Mann-Whitney test between females and males for each spindle characteristic is also reported. The whole cohort is used (phase 1 & 2, 180 subjects).

# Spindle dominant frequency for gold standard and each detector

| | Dominant oscillation frequency Hz | | | | | |
|---|---|---|---|---|---|---|
| Age | Younger | Older | P | Females | Males | P |
| GS | 13.30(0.57) | 13.11(0.79) | 0.106 | 13.28(0.68) | 13.17(0.68) | 0.0456 |
| a2 | 13.17(0.53) | 13.00(0.65) | 0.0382 | 13.27(0.53) | 13.00(0.60) | 0.000496 |
| a3 | 13.23(0.41) | 13.13(0.48) | 0.389 | 13.28(0.40) | 13.10(0.47) | 0.0243 |
| a4 | 13.23(0.43) | 13.07(0.44) | 0.0668 | 13.26(0.42) | 13.07(0.43) | 0.00256 |
| a5 | 13.40(0.38) | 13.35(0.39) | 0.433 | 13.43(0.35) | 13.27(0.38) | 0.00108 |
| a7 | 13.30(0.44) | 13.22(0.50) | 0.361 | 13.32(0.45) | 13.25(0.47) | 0.0784 |
| a8 | 13.24(0.40) | 13.09(0.45) | 0.0521 | 13.24(0.42) | 13.08(0.41) | 0.00632 |
| a9 | 13.31(0.51) | 13.23(0.64) | 0.526 | 13.32(0.57) | 13.24(0.56) | 0.0764 |

**Supplementary Table 7.** Spindle dominant frequency by-subject of the Gold Standard (GS) and the automated detectors (a2-a9) for young, old subjects, females and males. The median (and standard deviation) spindle frequency across subjects are reported. The p value (*P*) of the Mann-Whitney test between young and old groups and females and males is also reported. The whole cohort is used (phase 1 & 2, 180 subjects). A Group Consensus Threshold (*GCt*) =0.2 is used for the GS of the younger subjects (phase 1, 180 subjects) and a GCt=0.35 is used for GS of the older subjects (phase 2, 100 subjects).

# Correlation between gold standard and each detector for each spindle characteristic in phase 2

| Phase 2 | | | | | | | | |
|---|---|---|---|---|---|---|---|---|
| Correlation coefficient r² with the Gold Standard (*GS*) | | | | | | | | |
| Detectors | a2 | a3 | a4 | a5 | a7 | a8 | a9 | axAvg |
| Density (spm) | 0.27 | 0.51 | 0.33 | 0.43 | 0.83 | 0.04⁺ | 0.88 | 0.47 |
| Duration (s) | 0.23 | 0.03⁺ | 0.04⁺ | 0.02⁺ | 0.10 | 0.04⁺ | 0.12 | 0.08 |
| Amplitude (µV) | 0.76 | 0.86 | 0.84 | 0.86 | 0.88 | 0.81 | 0.84 | 0.84 |
| Frequency (Hz) | 0.24 | 0.58 | 0.56 | 0.47 | 0.46 | 0.41 | 0.53 | 0.46 |

**Supplementary Table 8.** Correlation coefficient r² between Gold Standard (*GS*) and automated detectors (*a2-a9*) for the spindle density, average duration, amplitude and frequency by-subject in phase 2. The mean $r^2$ across detectors is also reported (*axAvg*). The correlation coefficient p-value was significant (< 0.05) for each detector except for the spindle density of a8 and the average spindle duration of a3, a4, a5 and a8 shown with the ‘⁺’ marker. The phase 2 is used (older 80 subjects).

## The average spindle density, duration, amplitude and frequency for each detection method

| | Density spm | | | Duration s | | | Amplitude µV | | | Frequency Hz | | |
|---|---|---|---|---|---|---|---|---|---|---|---|---|
| | Mean | STD | P | Mean | STD | P | Mean | STD | P | Mean | STD | P |
| GS | 3.8 | 2.4 | NA | 0.78 | 0.15 | NA | 30 | 7 | NA | 13.21 | 0.68 | NA |
| $GC_{re}$ | 3.9 | 2.4 | 0.74 | 0.8 | 0.14 | 0.14 | 34 | 7 | 6.50E-06 | 13.28 | 0.53 | 0.46 |
| $GC_{ne}$ | 4.2 | 2.2 | 0.11 | 0.84 | 0.15 | 0.0012 | 33 | 7 | 0.0011 | 13.18 | 0.61 | 0.73 |
| a2 | 0.9 | 0.7 | 8.60E-38 | 1.15 | 0.29 | 1.60E-33 | 43 | 12 | 9.50E-30 | 13.07 | 0.59 | 0.037 |
| a3 | 6.7 | 2.1 | 3.60E-25 | 0.56 | 0.08 | 4.70E-43 | 28 | 8 | 0.0075 | 13.16 | 0.44 | 0.24 |
| a4 | 3.8 | 1.3 | 0.54 | 0.67 | 0.07 | 1.10E-15 | 32 | 10 | 0.045 | 13.13 | 0.44 | 0.11 |
| a5 | 3.3 | 1.2 | 0.16 | 0.53 | 0.07 | 1.20E-48 | 33 | 10 | 0.002 | 13.34 | 0.38 | 0.065 |
| a7 | 3.9 | 2.1 | 0.49 | 0.85 | 0.18 | 6.30E-06 | 29 | 7 | 0.33 | 13.26 | 0.46 | 0.58 |
| a8 | 6.9 | 1 | 2.30E-34 | 0.73 | 0.14 | 0.0018 | 28 | 10 | 0.019 | 13.15 | 0.42 | 0.18 |
| a9 | 4.8 | 3.1 | 0.0036 | 1.15 | 0.15 | 2.00E-49 | 28 | 6 | 0.11 | 13.28 | 0.57 | 0.52 |

**Supplementary Table 9.** The average spindle density, duration, amplitude and frequency by-subject for the Gold Standard (*GS*), each detector (a2-a9) and the Group Consensus of researchers ($GC_{re}$) and non-experts ($GC_{ne}$). The mean and standard deviation (*STD*) characteristics are reported. The Mann-Whitney-Whitney p value (*P*) test of the difference with the *GS* is also reported. The whole cohort is used (phase 1 & 2, 180 subjects) except for the $GC_{re}$ and $GC_{ne}$ where only the young subjects have been scored (phase 1, 100 subjects). The Group Consensus Threshold (*GCt*) used for the GS is 0.2 for phase 1 and 0.35 for phase 2. The *GCt* =0.15 for the $GC_{re}$ and 0.3 for the $GC_{ne}$.

# An example of a practice trail in the MODA web interface

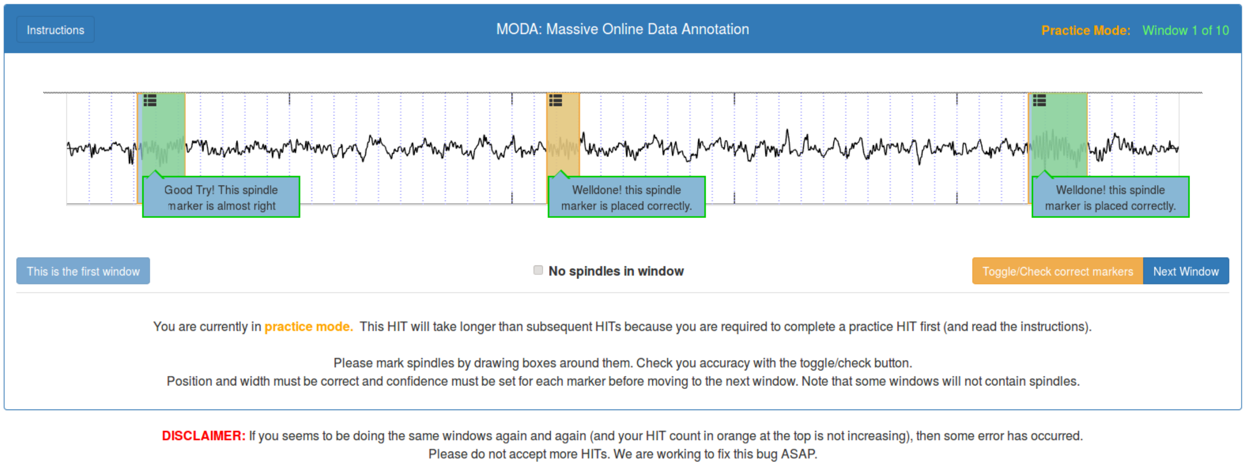


**Supplementary Figure 3.** An example of a practice trail to score spindles with feedback on the MODA (Massive Online Data Annotation) website.
